# Quantitative and sensitive detection of alpha fetoprotein in serum by a plasmonic sensor

**DOI:** 10.1515/nanoph-2022-0428

**Published:** 2022-10-24

**Authors:** Yang Xiong, Huatian Hu, Tianzhu Zhang, Yuhao Xu, Fei Gao, Wen Chen, Guangchao Zheng, Shunping Zhang, Hongxing Xu

**Affiliations:** The Institute for Advanced Studies, Wuhan University, Wuhan 430072, China; School of Physics and Technology, Center for Nanoscience and Nanotechnology, and Key Laboratory of Artificial Micro- and Nano-structures of Ministry of Education, Wuhan University, Wuhan 430072, China; Physics Teaching and Research Section, Zunyi Medical Univrsity, Zunyi 563003, China; Laboratory of Quantum and Nano-Optics, Ecole Polytechnique Fédérale de Lausanne, Lausanne CH-1015, Switzerland; Key Laboratory of Materials Physics of Ministry of Education, School of Physics and Microelectronics, Zhengzhou University, Zhengzhou 450052, China; Wuhan Institute of Quantum Technology, Wuhan 430206, China; School of Microelectronics, Wuhan University, Wuhan 430072, China

**Keywords:** biomarkers, nanoparticle-on-mirror, quantitative detection, surface plasmon polaritons, SERS

## Abstract

Quantitative molecular detection based on surface-enhanced Raman spectroscopy (SERS) is still a great challenge because of the highly nonuniform distribution of the SERS hot spots and the nondeterministic spatial and spectral overlap of the analyte with the hot spot. Here, we report a nanoparticle-on-mirror plasmonic sensor excited by surface plasmon polaritons for quantitative SERS detection of alpha fetoprotein in serum with ultrahigh sensitivity. The uniform gaps between the nanoparticles and gold film and the alignment of the gap modes relative to the excitation electric field endow this substrate with a uniform and strong SERS enhancement. The limit of detection reaches 1.45 fM, 697 times higher than that under normal excitation and 7800 times higher than a commercial enzyme-linked immunosorbent assay kit. This approach offers a potential solution to overcome the bottleneck in the field of SERS-based biosensing.

## 1 Introduction

Surface-enhanced Raman spectroscopy (SERS) is a powerful analytical tool with fingerprint information of target molecules [1–4]. It can achieve single molecules sensitivity [5–8], much more sensitive than the other detection techniques, e.g., fluorescence [9], surface plasmon resonance [10], electrochemical [11], and dark-field microscopy [12]. However, quantitative detection based on SERS is still a big challenge because the SERS enhancement is mainly originated from the electric field enhancement which is very sensitive to the shape, size, gap distance between nanostructures [13, 14], and the polarization and wavelength of incident light [15, 16], etc. Moreover, previous studies have shown that 85% of SERS signal is contributed by ∼6% of molecules that are located around the most intense hot spots [17]. This large nonuniformity indicates the low quantitative performance for molecular detection using traditional SERS substrates, hampering its biosensing application towards practical disease diagnosis, etc.

To improve the quantitative capability of SERS, different strategies have been proposed to generate uniform hot spots and enrich the analytes within the hot spot [18–22]. For example, intergap and intragap nanostructures are an excellent SERS substrate in the detection of small molecules [23–32]. The ordered nanostructures or uniform gap render these substrates reproducible. The internal standard method is another strategy to improve quantitative capability [33–35]. The Raman signal of internal standard molecules is used to calibrate the intensity fluctuations. In addition, eliminating abnormal hot sites can also improve the quantitative capability [36, 37]. Although these methods have shown their capability in quantitative SERS detection of small molecules with large Raman cross sections, their transfer to biomarker detection is still a challenge because most biomarkers have relatively low Raman cross sections and low binding affinity for the metal surface. The transfer from a proof-of-concept laboratory experiment to realistic environments with influence from other irrelevant molecules further lifts the requirement for the stability and reproducibility of the SERS substrate.

Here, we report a nanoparticle-on-mirror (NPOM) plasmonic sensor excited by surface plasmon polaritons (SPPs) to realize quantitative SERS detection of alpha fetoprotein (AFP), one biomarker for hepatocellular cancer. The direction of the electric field of SPPs excited by the Kretschmann configuration is normal to the gold film, which can excite the gap modes between the gold nanoparticle (AuNP) and the gold film well and provide uniform and strong electric field enhancement [38]. Based on these, we realize quantitative detection of AFP with a detection limit down to 1.45 fM. The sensitivity is 697 times higher than that under normal excitation and 7800 times higher than a commercial enzyme-linked immunosorbent assay (ELISA) kit. Our work offers a reliable method for quantitative and sensitive detection of disease biomarkers, which may promote the use of SERS in disease diagnosis and monitoring.

## 2 Results and discussion

**Figure 1:**
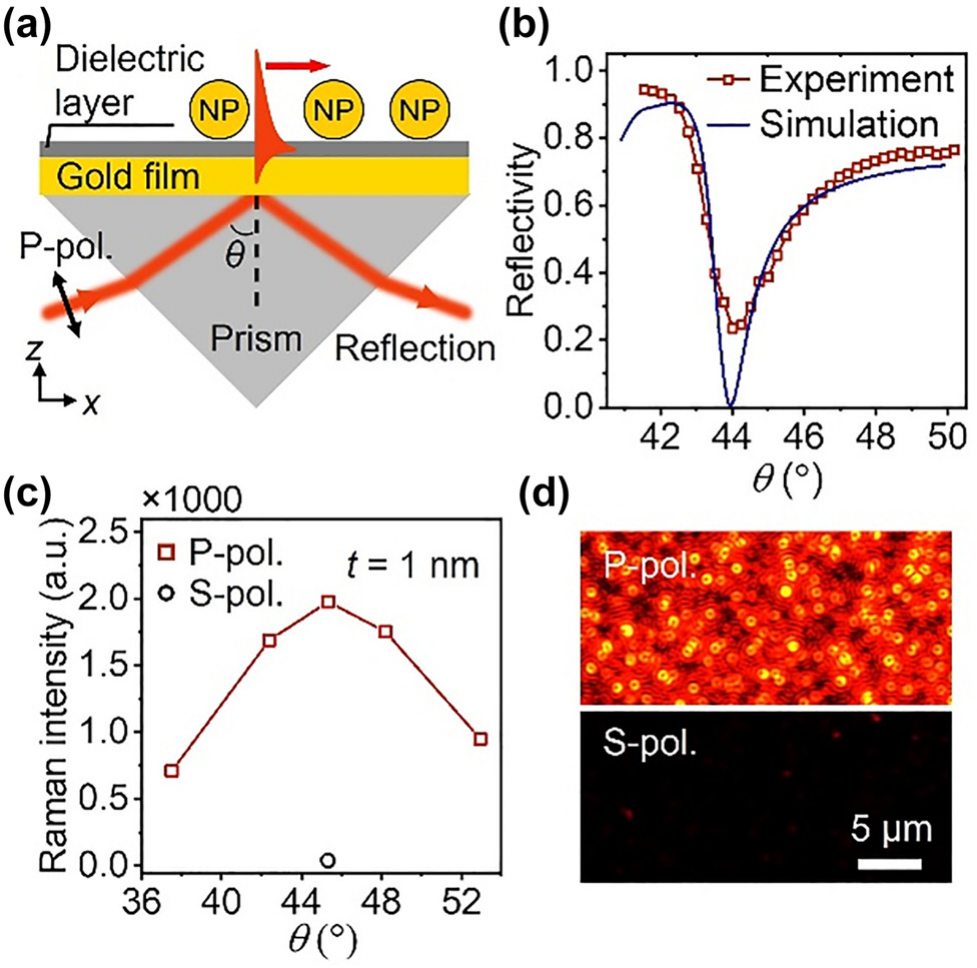
Exciting NPOM system by prism coupling. (a) Schematic of the NPOM excited by SPPs in the Kretschmann configuration. The thickness of the gold film is 45 nm, and the diameter of AuNP is 60 nm. The laser wavelength is 632.8 nm, and the thickness of dielectric layer is defined as *t*. (b) Measured and simulated reflection curves of a clean gold film under p-polarized light incident. (c) Plot of SERS intensity of 4-MBA molecules at 1075 cm^−1^ as a function of the incident angle. The thickness of Al_2_O_3_ layer is 1 nm. (d) Dark-field images of the NPOM substrate excited by p- (s-) polarized light at the angle of 45.32°.

The NPOM geometry [13, 28, 39], [40], [41], [42], [43], [44], [45], [46] is an appealing SERS substrate that can be prepared by immobilizing NPs on a metal film. Compared with single NPs or nanoparticle-on-dielectric substrates, NPOM can provide larger electric field enhancement due to the near-field coupling between the NP and the metal film [13, 40, 47]. The AuNP and its electromagnetic image in the gold film interact in a way similar to a NP dimer. The near field coupling strength between the NP and the metal film depends on the gap distance between them, approximately in an exponential manner [13]. Strong field enhancement occurs in the gap when the vertical orientation of the dipole moment of the bonding dipole–dipole mode in the NPOM is excited by a vertical electric field [38, 48]. SPPs excited by prism coupling can effectively stimulate this mode. Figure 1a shows the schematic of NPOM substrate excited by SPPs with the Kretschmann configuration. The incident light is focused onto the surface of the gold film by a convex lens (*f* = 20 cm) in front of the one side of the prism. Due to the hydrophobicity of the gold film, it is difficult for NPs to adsorb onto gold film directly. A dielectric layer of Al_2_O_3_ or protein absorbed on the gold film was used to immobilize NPs. Figure 1b shows the measured and simulated reflection curves for a clean gold film under p-polarized collimated light incident from the prism side. The resonance condition can be determined by scanning the incident angle of the laser, on which the light is effectively converted to SPPs on the gold surface and thereby shows minimal reflected intensity. The measured reflectivity decreases sharply at 44° and then increases again. The simulated reflectivity performed using the finite element method matches well with the experiment results. The simulated electric field distributions (Figure S1) indicate that the electric field near the metal–air interface is 8.24 times the excitation plane wave when the light incidents at the SPR angle, in accordance with the reflectivity measurement. This field enhancement is the consequence of the field confinement associated with the free electrons at the metal surface.

The SPR angle is very sensitive to the refractive index of the medium on the surface of the gold film, and it will increase when the medium is changed from air to Al_2_O_3_ or protein layer. We use 1-nm-thick Al_2_O_3_ layer as an example to find the SPR angle of the NPOM substrate to obtain the largest SERS enhancement. The NPOM substrate was prepared by electrostatic self-assembly. 3-aminopropyltriethoxysilane molecules absorbed on the Al_2_O_3_ layer was used to immobilize NPs. 4-mercaptobenzoic acid (4-MBA) molecules with a concentration of 10^−6^ M, absorbed on the surface of gold film, were used as Raman probes. Figure 1c shows the plot of SERS intensity at 1075 cm^−1^ as a function of the incident angle. The Raman peaks at 1075 and 1585 cm^−1^ are the characteristic peaks of 4-MBA. The largest SERS intensity was collected at the angle of 45.32° under p-polarized light excitation, which is slightly larger than the SPR angle of the clean gold film. Under s-polarized light incident, almost no Raman signal was collected, indicating that s-polarized light can’t excite the SPPs. This phenomenon can be also inferred from the dark-field images of AuNPs, as shown in Figure 1d. Under p-polarized light excitation, the scattering of the AuNPs is very strong, indicating that a lot of light was coupled to the NPOM, but under s-polarized light incident, the scattering is very weak. The AuNPs appear as doughnut shapes in the dark-field image, showing that the radiation from each AuNP is associated with the bonding dipole-dipole mode perpendicular to the gold film. The measured and simulated dark-field scattering spectra and electric field distributions of the NPOM are shown in Figure S2. The dark-field scattering spectra show that the bonding dipole-dipole peaks of the NPOM locate at 660 nm, matching well with the laser and Raman lines. Electromagnetic calculations also find that under prism excitation at an angle of 45.32°, the NPOM provides the largest electric field enhancement (Figure S3).

**Figure 2:**
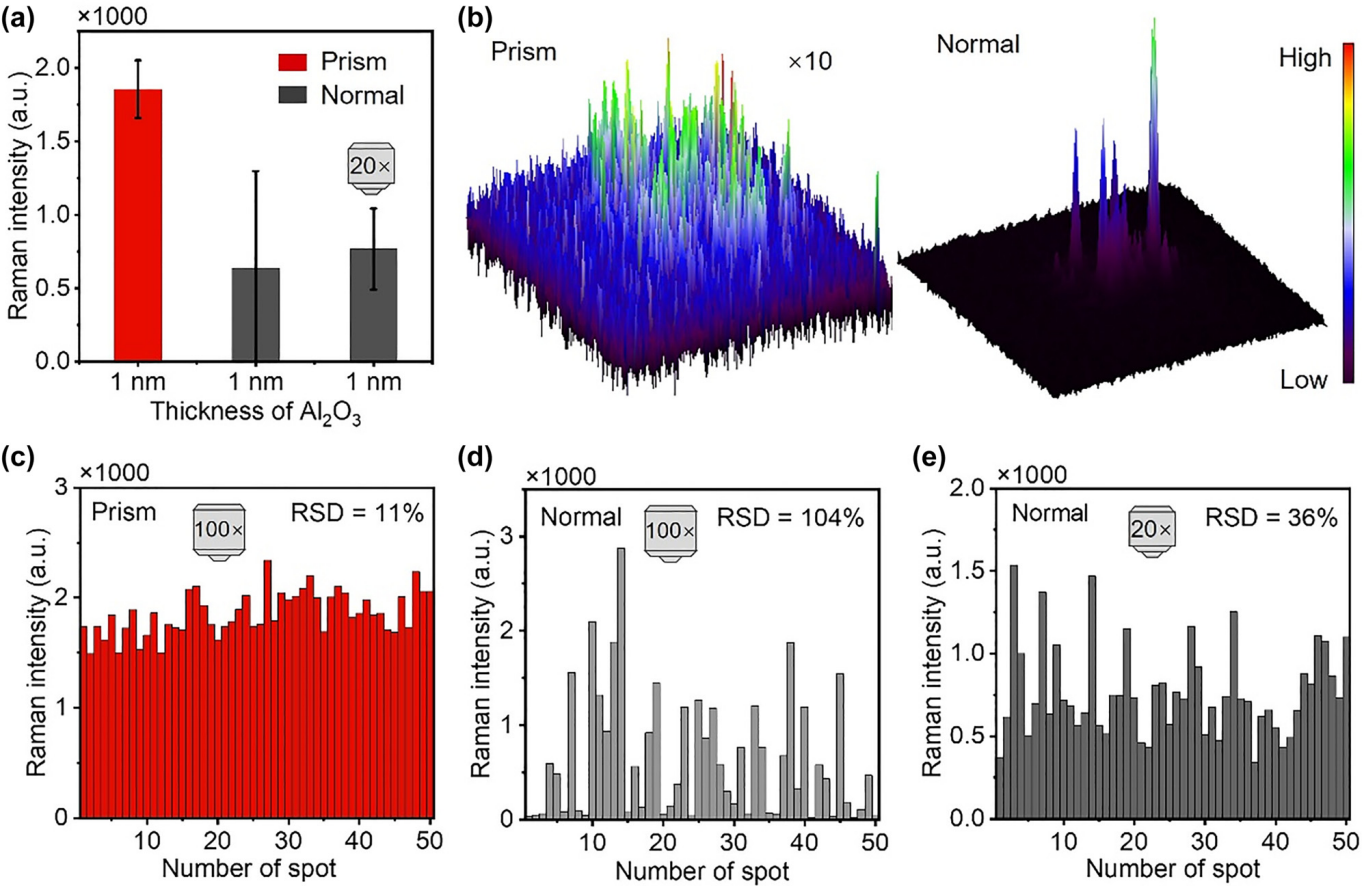
Comparing the uniformity of SERS enhancement. (a) SERS intensities of 4-MBA molecules at 1075 cm^−1^ under prism excitation and normal excitation. Error bars indicate the standard deviations of 50 measurements. (b) Raman images at 1075 cm^−1^ under prism (left) and normal excitation (right). (c–e) SERS intensities at 1075 cm^−1^ of 50 SERS spectra recorded under prism excitation (c), normal excitation with 100× objective (d) and 20× objective (e).

Figure 2a shows the SERS intensities of 4-MBA molecules at 1075 cm^−1^ under prism excitation at the angle of 45.32° and normal excitation. The setups for SERS measurement under prism and normal excitation are shown in Figure S4. A 100× objective (NA = 0.9) was used to collect the Raman signal in the two excitation configurations. To reveal the effect of numerical aperture and spot size of the excitation laser, a low magnification objective (20×, NA = 0.45) was used under normal excitation. Under prism excitation, the SERS intensity is 2.92 times larger than that under normal excitation with 100× objective, even though the power density for normal excitation reaches 391.72 W/mm^2^, which is 1780 times larger than that prism excitation (0.22 W/mm^2^). This is due to the high excitation efficiency of the vertical SPPs near field for the bonding dipole-dipole mode in the NPOM system. From its doughnut shape of the emission pattern shown in dark-field image, optical reciprocity requires that this mode cannot be excited by a strictly normal incident plane wave. Therefore, under normal excitation in a typical optical microscope, the excitation efficiency is very low because only a small component of light is polarized normal to the surface of gold film after being focused by an objective [28]. The power density for normal incidence with 20× objective is 74.61 W/mm^2^. Though the collection area becomes larger, the SERS signal intensity is 2.42 times smaller than that prism excitation, shown in Figure 2a.

The uniform electric field enhancement can be reflected by Raman imaging. Figure 2b shows the Raman images of 4-MBA molecules at 1075 cm^−1^ under prism and normal excitation. The 100× objective was used to collect the Raman signal in both excitation configurations. Under prism excitation, the SERS intensities in the excitation region are uniform, indicating uniform enhancement in the area. However, under normal excitation in the same area, there are a few sites with strong Raman signals, and the SERS intensity of each hot spot varies dramatically, revealing the nonuniform enhancement. This phenomenon can be also observed in other regions (Figure S5). The uniform signal intensity under prism excitation is attributed to the following three aspects. (i) The uniform size of AuNPs and the uniform gap distance between the AuNPs and gold film, which ensure a similar resonance peak of the gap plasmon mode. (ii) The alignment of the direction of the electric field relative to the axis of the NPOM, which ensures a similar excitation efficiency of different NPOMs. (iii) The distribution of hot spots is uniform because the AuNPs are well dispersed on the gold film, which can be inferred from the dark-field image and scanning electron microscope image of NPOM substrate (Figure S6a–c). Although there is a small amount of AuNPs aggregates (Figure S6d–f), the gap plasmon resonances between NPs cannot be efficiently excited under prism excitation. However, under normal excitation, these aggregates can generate highly nonuniform hot spots because of the random orientation of inter-particle axes of the aggregates with respect to the laser polarization and together with the uncontrollable gap distances between the AuNPs.

In addition to Raman imaging, the relative standard deviation of SERS intensity could be also used to reflect the uniformity of the electric field enhancement. Under prism excitation, the relative standard deviations of SERS intensities at 1075 cm^−1^ are 11%. However, under normal excitation, the relative standard deviation reaches 104%, shown in Figure 2c and d. The relative standard deviation is calculated from the intensity data of 50 spectra which were recorded along a line in the center of each sample. Furthermore, the relative standard deviation is still 36% under normal excitation with the 20× objective, shown in Figure 2e. This indicates that under prism excitation, the spectral reproducibility is better than that under normal excitation since these data were collected from the same substrate. In addition to the reasons discussed above, under prism excitation, the over or under focus in the microscope only affects the collection signal because the size of the excitation laser spot focus by the convex lens is relatively large (up to 180 × 250 µm^2^) and insusceptible to the drift of the focus. However, under normal excitation, both over and under focus can significantly affect the size of the excitation laser spot as well as the signal collection; especially for a high NA objective. This results in larger signal fluctuation, as shown in Figure S7.

**Figure 3:**
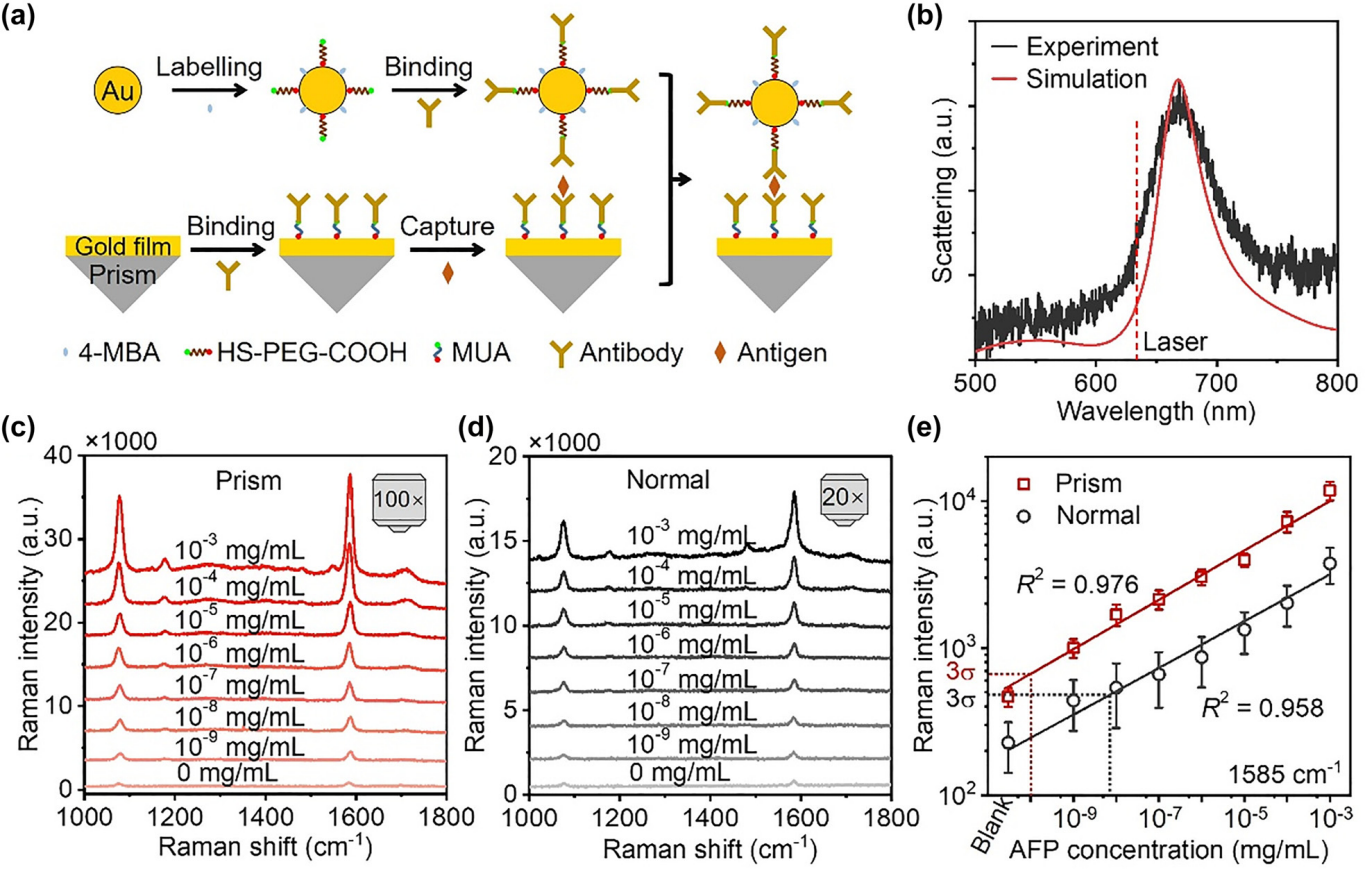
SERS-based immunoassay for sensitive detection of AFP. (a) Schematic of the process of preparing immune NPOM substrate. (b) Measured and simulated dark-field scattering spectra of the immune NPOM. (c, d) SERS spectra of the immune NPOM substrates with the concentration of AFP ranging from 10^−3^ to 10^−9^ mg/mL under prism (c) and normal excitation (d). (e) Plots of SERS intensities at 1585 cm^−1^ as a function of the concentration of AFP. Error bars indicate the standard deviations of 50 measurements. *σ* represents the relative standard deviation of the SERS intensities of the blank sample.

Based on the above analysis, we use this plasmonic sensor to realize quantitative detection of AFP. As the Raman signal of most protein biomarkers is intrinsically low and can be easily overwhelmed by the background signal, hence, labeling the NPs with Raman molecules is required [49, 50]. Figure 3a shows the process of forming immune NPOM substrate by three components, immune NPs, target antigen (AFP), and immune substrate. The detailed process is in Supporting Information. Al_2_O_3_ film is not used in the immunoassay because there is a dielectric layer (11-mercaptoundecanoic acid and antibody protein, etc.) inside the gap between the AuNPs and gold film. 11-mercaptoundecanoic acid can be absorbed on the gold film like Al_2_O_3_ film to form densely packed and well-ordered self-assembled monolayers [51]. Figure 3b shows the measured and simulated dark-field scattering spectra of the immune NPOM. The bonding dipole–dipole peak of the immune NPOM is at 668 nm. Through theoretical simulation, the thickness of the layer inside the gap of immune NPOM is about 1.45 nm, which is almost the same as the gap distance of the NPOM with 1-nm-thick Al_2_O_3_ and an extra 0.6-nm-thick 3-aminopropyltriethoxysilane discussed above. The electric field simulation result indicates that under prism excitation at the angle of 45.32°, the immune NPOM provides the largest enhancement (shown in Figure S8).

The Raman spectra of the immune NPOM substrate with the concentration of AFP ranging from 10^−3^ mg/mL to 10^−9^ mg/mL under the two excitation configurations were recorded, shown in Figure 3c and d. Under normal excitation, the 20× objective was used. The 100× objective was not used under normal excitation because the above analysis shows that the 100× objective is not suitable for quantitative detection. Strong Raman signals of 4-MBA molecules were detected in both excitation configurations, and the intensities decrease with the decrease of the concentration of AFP target. The SERS intensity at 1585 cm^−1^ under prism excitation is almost two times larger than that under normal excitation for each concentration, which attributes to the larger SERS enhancement under prism excitation.

Figure 3e shows the plot of the SERS intensity at 1585 cm^−1^ as a function of the concentration of AFP. 50 Raman spectra were recorded from two substrates for each concentration. Under prism excitation, the relationship between the concentration and SERS intensity can be described by log *I* = 0.168 log *C* + 4.504 with *R* ^2^ = 0.976, where the *I* and *C* represent the SERS intensity at 1585 cm^−1^ and the concentration of AFP. Under normal excitation, the calibration curve is fitted as log *I* = 0.159 log *C* + 3.978 with *R* ^2^ = 0.958. It’s obvious that under prism excitation, the calibration curve shows a better linear response in this broad dynamic range. The relative standard deviations of SERS intensity at 1585 cm^−1^ for the concentration of AFP ranging from 10^−3^ to 10^−9^ mg/mL are 10–19% for prism excitation and 27–47% for normal excitation, indicating a better spectral reproducibility of the former. The poor reproducibility under normal excitation is due to the different near-field coupling strengths between AuNPs in AuNP aggregates as discussed above. The formation of AuNP aggregates is random and inevitable in the physiological environment, resulting in uncontrollable gap distance between AuNPs (shown in Figure S9).

According to the definition of the limit of detection (LOD), *I*_LOD_ ≥ *I*_Blank_ + 3*σ* [49], the LODs are 100 fg/mL (1.45 fM) and 69.7 pg/mL (1010.14 fM) for prism and normal excitation, as shown in Figure 3e. The detection sensitivity under prism excitation is 697 times higher than that under normal excitation and 7800 times higher than a commercial ELISA kit (LOD, 0.78 ng/mL), NeoBioscience Technology Co., Ltd. The SERS intensity at 1585 cm^−1^ of the blank sample is small in both excitation configurations, indicating a small amount of nonspecific adsorption. Compared with the current biomarker detection methods, the sensitivity of this work is higher than the other detection methods and slightly lower than the best one based on SERS, shown in Table S1 [52–60]. However, although high sensitivity is achieved in those works, the quantitative capability is poor because of the poor reproducibility of SERS under normal excitation even if 20× objectives were used to excite and collect Raman signals. To verify the universality of this plasmonic sensor, we also detected prostate specific antigen. The SERS spectra and the plot of the SERS intensity as a function of the concentration are shown in Figure S10. SERS signals of samples with concentration ranging from 10^−3^ to 10^−9^ mg/mL are detected in the experiment, and the LOD is 180 fg/mL.

**Figure 4:**
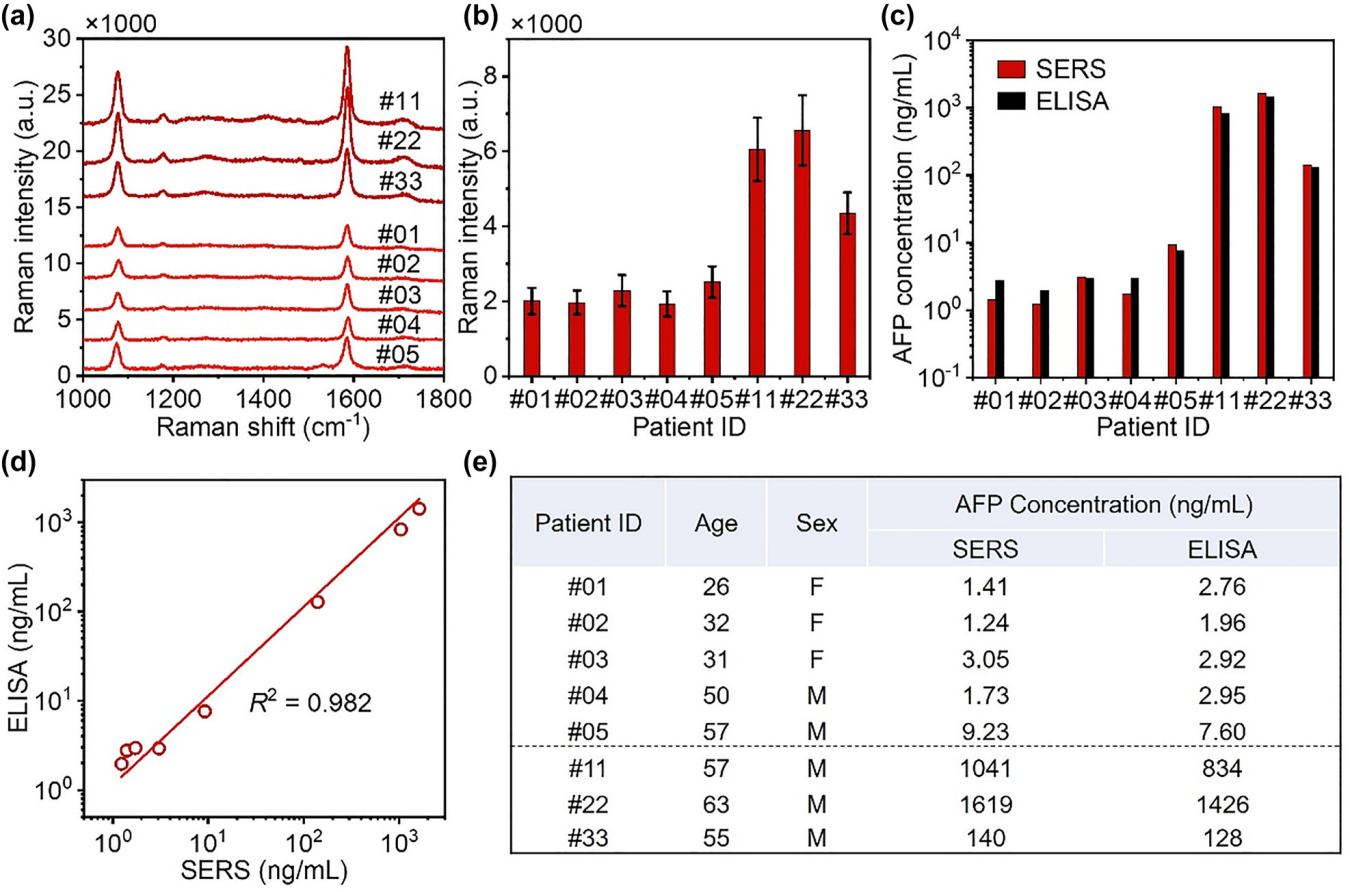
Quantitative detection of AFP in blood sample. (a) SERS spectra for serum samples (diluted 20-fold) from eight people (ID: #01, #02, #03, #04, #05, #11, #22, #33). (b) SERS intensities at 1585 cm^−1^ of AFP immunoassay for eight people. (c) AFP concentrations in the blood samples as determined by SERS and ELISA. (d) Plot showing the correlation between the concentration of AFP determined by SERS and ELISA. (e) Table summarizing the age, sex, and measured concentrations of AFP.

Following the successful demonstration, we set out to analyze eight blood samples from three patients and five healthy volunteers to determine the concentrations of AFP. Meanwhile, ELISA method was used to verify the reliability of this plasmonic sensor. Figure 4a shows the SERS spectra for serum samples from eight people, and the SERS intensities at 1585 cm^−1^ of AFP immunoassay from eight people are shown in Figure 4b. The SERS intensities for patient serum samples are much larger than that of healthy volunteers. The concentrations of AFP in the serum samples were quantified by the SERS intensities, shown in Figure 4c. The concentrations of AFP in the serum samples were larger than 100 ng/mL for patients and less than 10 ng/mL for healthy volunteers. AFP as a marker of liver cancer, when the concentration is greater than 20 ng/mL, it may be related to liver cancer [61]. The concentrations of AFP in serum samples quantified by the ELISA method are shown in Figure 4c. The calibration curve of ELISA was shown in Figure S11. The concentrations of AFP in serum samples determined by this plasmonic sensor showed excellent agreement with that determined by ELISA, shown in Figure 4d. The age, sex, and accurate concentrations quantified by the two methods are shown in Figure 4e. Two independent experiments were conducted for the detection of samples #01, #02, #03, #11, #22, #33 and #04, #05.

## 3 Conclusions

A SERS-based biosensor has been developed for ultrasensitive and quantitative detection of alpha fetoprotein in blood sample. The NPOM substrate excited by SPPs converts the traditional hot spots between NPs into hot spots generated by the gap between NPs and metal film, which endows the substrate with uniform and strong electric field enhancement. Raman images and spectral reproducibility indicate the uniform SERS enhancement under prism excitation. A good spectral reproducibility and ultralow detection limit down to 1.45 fM were achieved. This sensitivity is 697 times higher than that under conventional configuration and 7800 times higher than a commercial ELISA kit. This sensitivity and reproducibility enable the quantification of AFP in serum samples, with excellent accuracy in comparison with ELISA. This novel plasmonic sensor has significant potential applications in analytical chemistry, environmental pollution detection, food safety, and biomedical fields.

## Supplementary Material

Supplementary Material Details
