# Supplementary material for: Quantitative and sensitive detection of alpha fetoprotein in serum by a plasmonic sensor
Source: Nanophotonics. 2022 Oct 24;11(21):4821–9. doi: 10.1515/nanoph-2022-0428 (PMC11501717; doi:10.1515/nanoph-2022-0428)
Supplement: Supplementary file 1 — Supplementary Material Details [file j_nanoph-2022-0428_suppl_001.docx]

Supplementary Information for

Quantitative and sensitive detection of alpha fetoprotein in serum by a plasmonic sensor

Yang Xiong,^†a^ Huatian Hu,^‡a^ Tianzhu Zhang,^b^ Yuhao Xu,^b^ Fei Gao,^c^ Wen Chen,^d^ Guangchao Zheng,^e^ Shunping Zhang,^*,b,f^ and Hongxing Xu^*,a,b,f,g^

^a^The Institute for Advanced Studies, Wuhan University, Wuhan 430072, China.

^b^School of Physics and Technology, Center for Nanoscience and Nanotechnology, and Key Laboratory of Artificial Micro- and Nano-structures of Ministry of Education, Wuhan University, Wuhan 430072, China.

^c^Physics Teaching and Research Section, Zunyi Medical Univrsity, Zunyi 563003, China.

^d^Laboratory of Quantum and Nano-Optics, Ecole Polytechnique Fédérale de Lausanne, CH-1015 Lausanne, Switzerland.

^e^Key Laboratory of Materials Physics of Ministry of Education, School of Physics and Microelectronics, Zhengzhou University, Zhengzhou 450052, China.

^f^Wuhan Institute of Quantum Technology, Wuhan 430206, China.

^g^School of Microelectronics, Wuhan University, Wuhan 430072, China.

^†^Present address: College of Information Engineering, Hubei University of Chinese Medicine, Wuhan 430072, China.

^‡^Present address: Hubei Key Laboratory of Optical Information and Pattern Recognition, Wuhan Institute of Technology, Wuhan 430205, China.

**Part Ⅰ Methods**

**Materials**. AuNPs (60 nm) solution was purchased from BBI solutions. 4-mercaptobenzoic acid (4-MBA), 3-aminopropyltriethoxysilane (APTES), and 11-mercaptoundecanoic acid (MUA) were purchased from Sigma-Aldrich. Thiol polyethylene glycol acid (HS-PEG-COOH, MW 3400), thiol polyethylene glycol (mPEG-SH, MW 5000), 1-ethyl-3-(3-dimethylaminopropyl) carbodiimide hydrochloride (EDC), and N-hydroxysuccinimide (NHS) were purchased from Aladdin Chemistry Co., Ltd (Shanghai, China). Bovine serum albumin (BSA) and phosphate-buffered saline (PBS, 1×, PH 7.4) buffer solution were purchased from Beyotime. Antigen, antibodies, and ELISA kit were purchased from NeoBioscience Technology Co., Ltd. Deionized water (18.3 MΩ·cm) was used throughout the experiments. All reagents were of analytical reagent grade and used without further purification.

**Preparation of NPOM substrate by self-assembly.** Glass slides (1 × 1 cm^2^) were cleaned with acetone, alcohol, and deionized water for 10 minutes each, and dried with nitrogen. Then 0.6-nm-thick chromium film and 45-nm-thick gold film were evaporated on the glass slides through thermal evaporation deposition, and the evaporation rate was kept at 0.5 Å s^-1^.

AuNPs were immobilized onto the gold film by electrostatic self-assembly. Briefly, the prepared gold films were first immersed in 10^-6^ M 4-MBA solutions. After 2 h, the gold films were cleaned with deionized water and dried with nitrogen. Then, 1, 3, and 5-nm-thick Al_2_O_3_ layers were deposited on the gold films through atomic layer deposition at 120 °C. Subsequently, the gold films were immersed in 3% APTES solution for 1 h at room temperature, then ultrasonicated in alcohol for 5 minutes and dried with nitrogen. Next, 5 µL AuNPs solution was dropped onto the gold film. The diameter of the droplet is about 3 mm. After waiting for 30 s, the AuNPs solution on the surface of the gold film was blown away with a rubber suction bulb. The negative charged AuNPs will be bound to the positive charged gold film, and the density of AuNPs can be controlled by controlling the adsorption time, shown in Figure S12. In our experiment, the average distance between AuNPs is about 1 µm, and this particle density can provide the largest electric field enhancement.^[1]^

**SERS Immunoassay**. Preparation of immune NPs. The processes of preparing immune NPs and immune substrates follow our previous work.^[2]^ Briefly, 2.5 µL 10^-3^ M 4-MBA solution was added to 500 µL AuNPs solution and shaking for 15 min. Then, 30 µL 10 µM HS-PEG-COOH solution was added. After shaking for 25 min, 60 µL 10 µM mPEG-SH solution was added. The mPEG-SH molecules can effectively prevent the aggregation of AuNPs in the biological environment.^[3]^ After shaking for 12 h, the mixture was centrifuged at 6000 rpm for 6 min. The supernatant was removed, and the sediment was resuspended in 500 µL PBS solution. To active the carboxyl of HS-PEG-COOH molecules for antibody binding, 5 µL 50 mM EDC and 5 µL 10 mM NHS solutions were added into the AuNPs solution. After 1 h, the mixture was centrifuged at 6000 rpm for 6 min, and the sediment was resuspended in 500 µL PBS solution. Next, 12.5 µL 1 mg/mL antibody (labeling) solution was added, and the mixture was incubated at 37 °C for 2 h. Then the excess antibodies were removed by centrifugation at 6000 rpm for 6 min. Finally, 10 µL 1% BSA solution was added to avoid nonspecific absorption, and then the mixture was stored at 4 °C for future use.

Preparation of immune substrate. First, the prepared gold films were immersed in 1 mL 10^-3^ M MUA solution. After 12 h, the gold films were rinsed with deionized water. To active the carboxyl of MUA for antibody binding, the gold films were immersed in 500 µL 50 mM EDC and 500 µL 10 mM NHS solutions for 1 h, and then rinsed with PBS and deionized water three times each. Next, 20 µL 1 mg/mL antibody (coating) solution was dropped onto the gold film and incubated at 37 °C for 2 h. After that, the gold films were rinsed with PBS and deionized water three times each. Then, 35 µL 1% BSA solution was dropped onto the gold film. After 1 h, the gold films were rinsed with PBS and deionized water three times each for future use.

Preparation of immune NPOM substrate. The prepared immune substrates were immersed in 900 µL antigen solution with concentrations ranging from 10^-3^ to 10^-9^ mg/mL then to 0 mg/mL or serum samples and incubated at 37 °C for 2 h. Then, the gold films were rinsed with PBS and deionized water three times each. Next, 30 µL immune NPs solution (AuNPs decorated with 4-MBA molecules and antibodies) was dropped onto the gold film and incubated at 37 °C for 2 h. Finally, the gold films were rinsed with PBS and deionized water five times each and dried with nitrogen for measurement.

Assays of clinical serum samples. Clinical serum samples were collected from Zunyi Medical University Affiliated Hospital and Hubei University of Chinese Medicine. This clinical study was approved by the Medical Ethics Committee of the hospital (No. 2022/01-9), and consent documents were also obtained from all patients. All the serum samples were stored at -20 °C and diluted 20 times with PBS in the SERS-based immunoassay. The concentrations of AFP for six clinical serum samples were determined using the proposed SERS-based immunoassay protocol and the commercially available ELISA kit, NeoBioscience Technology Co., Ltd. Finally, the SERS-based assay results were compared with those determined by the ELISA kit.

**Optical Characterization**. Dark-field scattering spectra were recorded on a spectrometer (Renishaw, inVia). Briefly, unpolarized light from a halogen lamp was directed through a commercial illuminator (Olympus BX51) with a dark-field module and focused onto the sample by a 100× objective (Olympus, NA = 0.9). The scattering light was collected by the same objective and directed onto the spectrometer by a flip mirror. The integration time is 10 s.

Raman spectra were recorded on the same spectrometer. The setup for SERS measurements is shown in Figure S4. For normal excitation, the excitation laser (wavelength 632.8 nm) was focused by the 100× objective or a 20× objective (Olympus, NA = 0.45) onto the sample. The scattering light was collected by the same objective and directed onto the spectrometer by a flip mirror. The laser power was 1.23 mW and 5.86 mW for the 100× and 20× objectives, and the diameter of the laser spot was 2 µm and 10 µm. The integration time was 10 s. For prism excitation, the excitation laser passed through a right-angle prism (refractive index 1.5168) and was focused onto the sample through a convex lens (ƒ = 20 cm). Immersion oil (Olympus, refractive index 1.518) was dropped between the substrate and prism in the measurement. The scattering light was collected by the 100× objective and directed onto the spectrometer by a flip mirror. The laser power was 7.80 mW, and the size of the laser spot was about 180 × 250 µm^2^. The collection area was set to ~ 12.5 × 12.5 µm^2^, and the integration time was 10 s. 50 Raman spectra were collected along a line at the center of each sample, and the spacing between two adjacent spots is ~30 μm. Note that in Figure S7, the SERS intensities were measured under only 0.12 mW laser power for normal excitation with 100× objective, because continuous strong laser irradiation will lead to light-induced damage and Raman signal fluctuation. For Raman imaging shown in Figure 2b, the laser power was 7.80 mW and 12.08 mW for prism and normal excitation, and the integration time was 30 s.

**Simulations.**

Full-wave simulation was performed via COMSOL Multiphysics 5.2a. When calculating the reflectivity and electric field distribution of the prism-chromium/gold layer substrate, 2D model was used for convenience. The refractive index of the prism was 1.5168, while the those of chromium and gold were taken from the Johnson and Christy.^[4]^ The prism is considered to be infinitely thick while the thicknesses of chromium and gold are 0.6 nm and 45 nm, respectively. The enhancement of the electric field was calculated by the electric field ***E*** normalized by the incident light ***E***_0_. When calculating the field enhancement of an SPPs-excited NPOM with a Al_2_O_3_ layer, 3D model was built and the Al_2_O_3_ layer (thickness *t* = 1, 3, 5 nm) was inserted between gold film and 60 nm AuNP. An extra 0.6-nm-thick dielectric layer was added to the alumina to account for the APTES adhere layer. The excitation port was activated in the bottom facet (prism) and the structure was excited by an oblique (45.32˚) p-polarized plane wave from the prism side. Therefore, the electric enhancement was defined as the electric field ***E*** in the NPOM nanogap normalized by incident ***E***_0_ in the prism. When calculating the scattering of the NPOM structure, the scattered field was obtained by a conventional two-step model. In the first step, the background field without NP was simulated and then the electric field distribution was transferred to the second step as a background excitation. The scattering was finally calculated by surface integrating the Poynting vector of the scattered field over solid angles covering the numerical aperture of the objective (NA = 0.9). When calculating the field enhancement and the scattering of an SPPs-excited the immune NPOM, the thickness of the dielectric layer inserted between gold film and AuNP was *t* = 0.85 nm. An extra 0.6-nm-thick dielectric layer was added to the surface of AuNP to account for the protein and HS-PEG-COOH molecule layer.

**Part Ⅱ Supporting figures**

**Section 1 Electric field distributions near the metal-air interface**

For a clean gold film under prism incidence, at different incident angle, the electric field enhancement is different. The largest enhancement can be obtained when light incidents at the SPR angle.

**
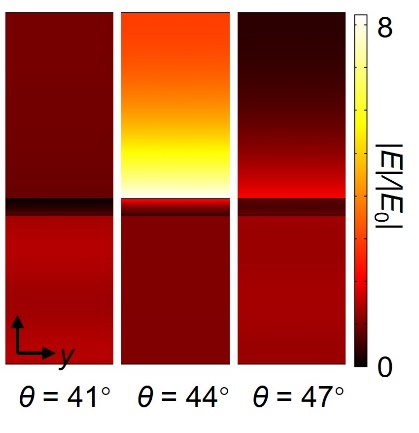
**

Figure S1 Electric field distributions near the metal-air interface at the angle of 41˚, 44˚, and 47˚.

**Section 2** **Dark-field scattering spectra and electric field distributions of NPOM**

The surface plasmon resonance wavelength matches well with the excitation laser wavelength, which can realize electric field enhancement.


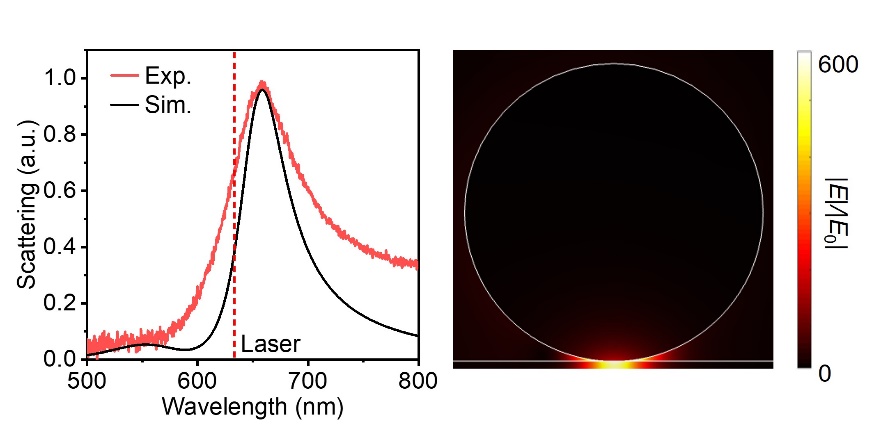


Figure S2 (a) Measured and simulated dark-field scattering spectra of NPOM with 1-nm-thick Al_2_O_3_ spacer. (b) Electric field distributions of NPOM with 1-nm-thick Al_2_O_3_ spacer, illuminated by a plane wave excitation incident from the prism with an incident angle of 45.32˚.

**Section 3.** **Electric field distribution of the NPOM with 1-nm-thick Al_2_O_3_ spacer**

Simulated electric field distributions indicate that under prism excitation, the largest electric field enhancement of the NPOM with 1-nm-thick Al_2_O_3_ spacer is obtained when light incident at the angle of 45.32˚.


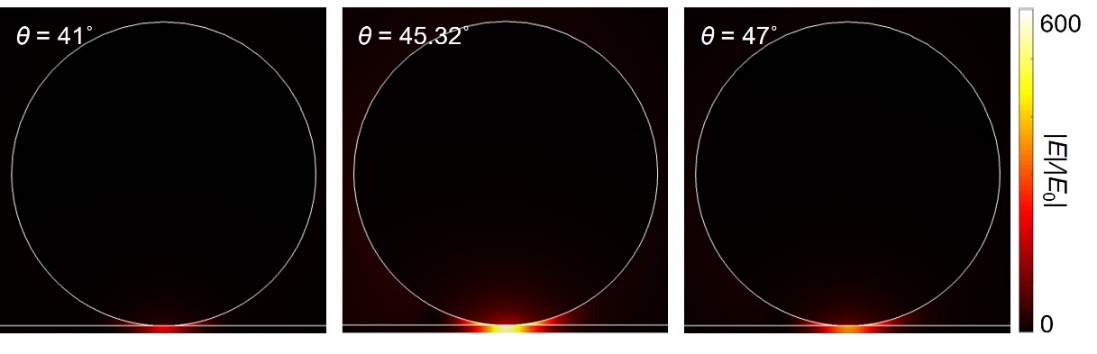


Figure S3 Electric field distributions of NPOMs with 1-nm-thick Al_2_O_3_ spacer under prism excitation at the angle of 41˚, 45.32˚, and 47˚.

**Section 4. Experimental setups**

Figure S4a shows the experimental setup for normal excitation. A 632.8 nm laser is incident along the normal direction of the sample and focused onto the sample by an objective. The Raman signal is excited and collected by the same objective. Figure S4b shows the experimental setup when the excitation laser is incident from the prism. The Raman signal is collected by the same objective as for normal excitation.


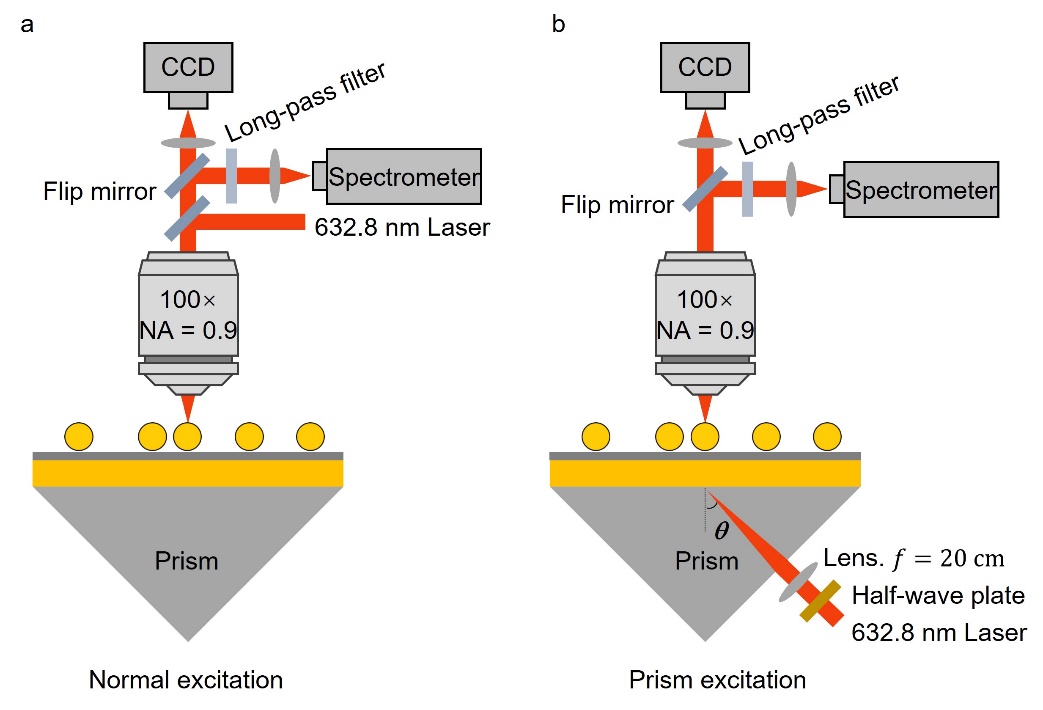


Figure S4 Experimental setups for SERS measurements for normal excitation (a) and prism excitation (b).

**Section 5 Raman imaging**

Raman imaging was performed at 1075 cm^-1^ under prism and normal excitation in the same area. Under prism excitation, the SERS intensities in the excitation area are uniform, indicating uniform electric field enhancement in the large area. However, under normal excitation, the SERS intensities vary dramatically in the same area.


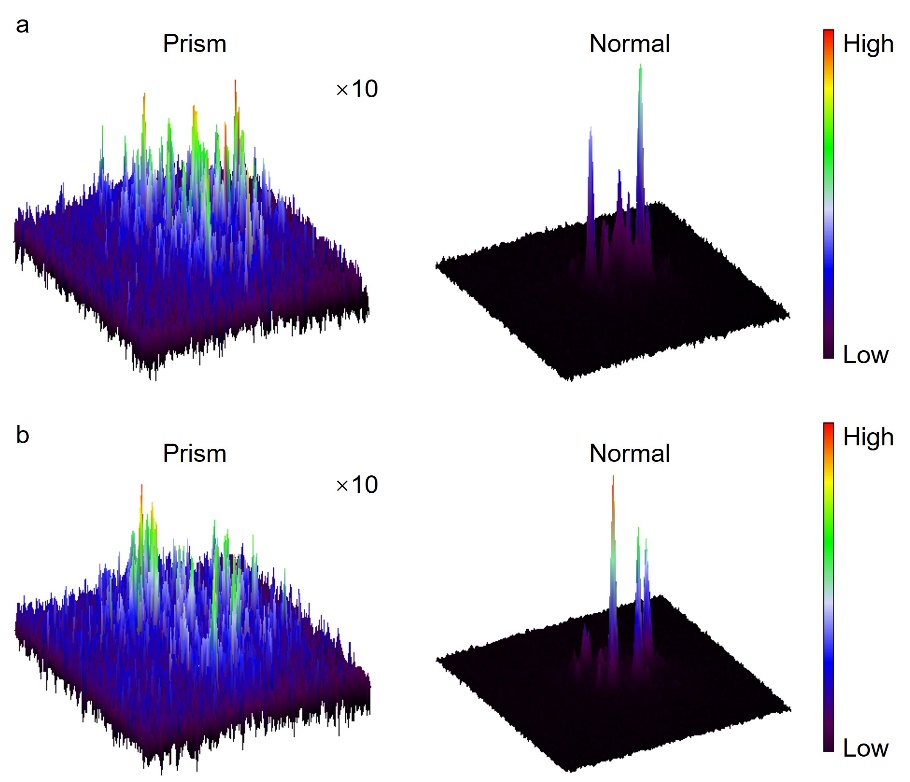


Figure S5 Raman images under prism excitation (left) and normal excitation (right) at two same positions, position 1 (a) and position 2 (b).

**Section 6 Dark-field image and scanning electron microscope image of the NPOM substrate**

Typical regions of the NPOM substrate in shown in Figure S6. AuNP is very bright in the dark-field image due to the superradiative nature of the bonding dipole-dipole mode in NPOM system. The random distribution and a fraction of aggregation of nanoparticles can be clearly seen from the scanning electron microscope image.


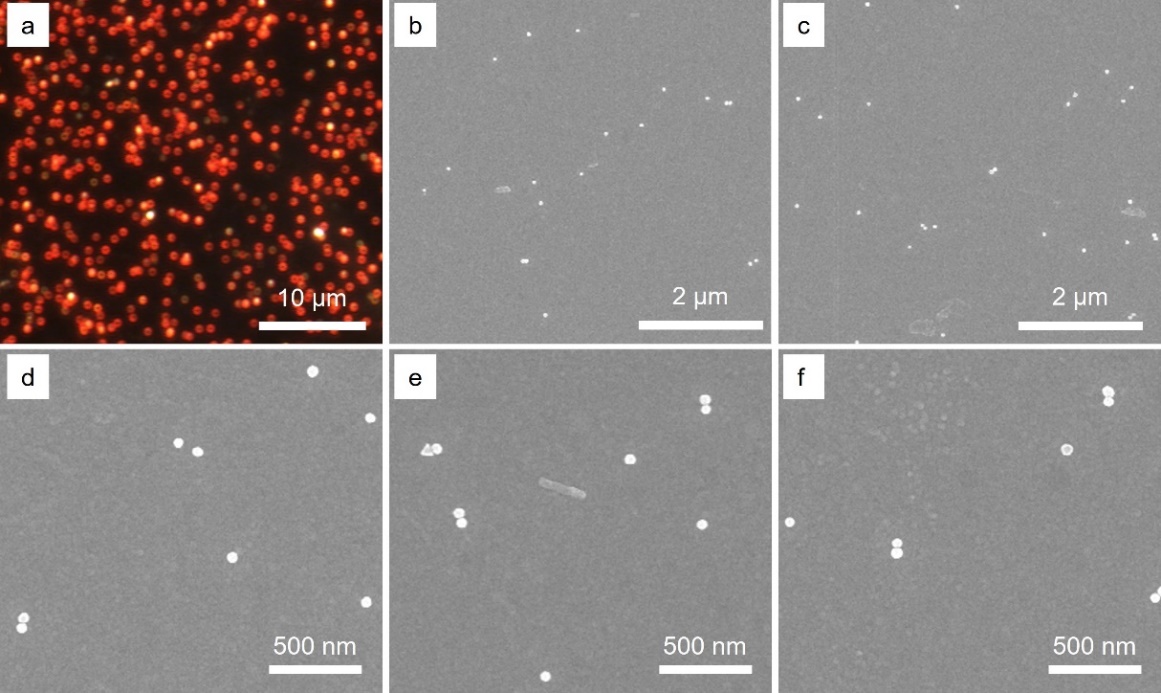


Figure S6 (a) Dark-field image of the NPOM substrate prepared by electrostatic self-assembly. (b-f) Scanning electron microscope images from different areas of the NPOM substrate.

**Section 7 Signal fluctuation caused by over or under focus**

Under incident from prism, over or under focus has little effect on the signal strength. By repeating the focusing 50 times and measuring the signal at one site, it is found that under incident from prism, the fluctuation of SERS intensity at 1075 cm^-1^ is smaller than that under normal excitation. This is because the 100× objective was used only in the collection optics in the former, but in both the excitation and collection optics in the later. The relative standard deviation of SERS intensity is 7.3% for incident from prism and is 9.7% for normal excitation (collection area 2×2 µm^2^). If the collection area expended to 12.5 × 12.5 µm^2^, the relative standard deviation of SERS intensity can get down to only 2.5% when incident from prism.


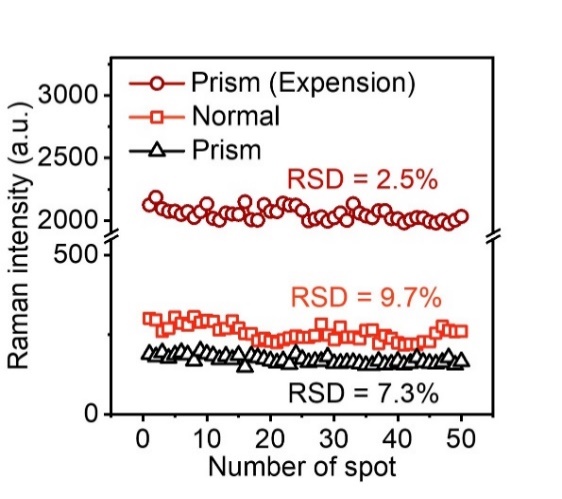


Figure S7 SERS intensities at 1075 cm^-1^ for 50 repeated measurements at one site for the NPOM substrate with 1-nm-thick Al_2_O_3_ spacer under the two excitation configurations. The 100× objective was used in both excitation configurations.

**Section 8 Electric field distribution of the immune NPOM configuration**

Simulated electric field distributions indicate that under prism excitation, the largest electric field enhancement for the immune NPOM is obtained when the incident angle is at 45.32˚. The thickness of the dielectric layer absorbed on the surface of AuNP is 0.6 nm.


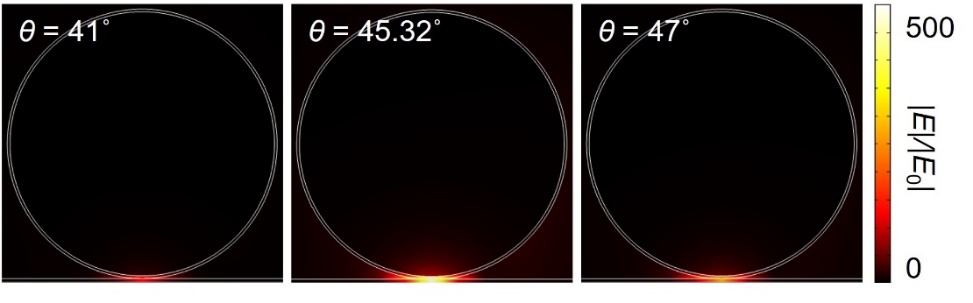


Figure S8 Electric field distributions of the immune NPOM configuration under prism excitation at the angle of 41˚, 45.32˚, and 47˚.

**Section 9 Scanning electron microscope image of the immune NPOM substrate**

The formation of AuNP aggregates is inevitable in the physiological environment, and the AuNPs are immobilized on the gold film randomly by immunoassay protocol, resulting in nonuniform distribution of the AuNP aggregates and uncontrollable gap distance between AuNPs. Figure S9 shows the scanning electron microscope images of the immune NPOM substrate with 10^-4^ mg/mL AFP. The distribution and aggregation of nanoparticles can be seen in the scanning electron microscope images.


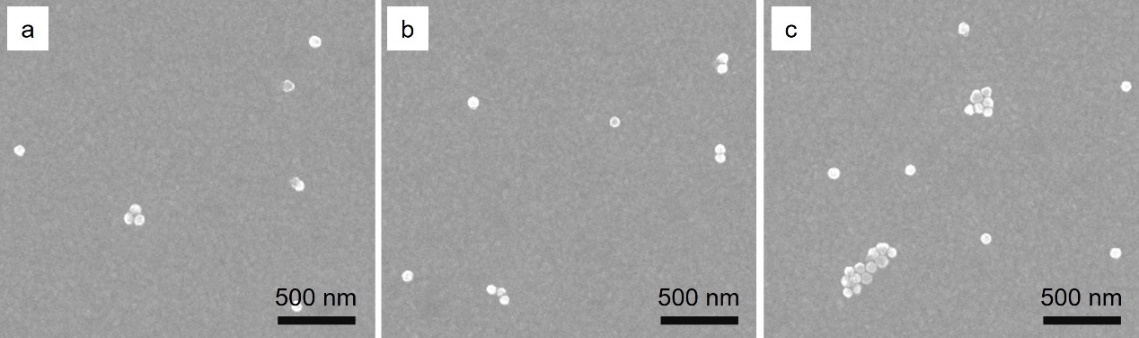


Figure S9 (a-c) Scanning electron microscope images of the immune NPOM substrate in different areas. The concentration of AFP is 10^-4^ mg/mL.

Table S1 Comparison of biomarkers detection based on various methods.

| Biomarker | Sensing principle | LOD | Reference |
| --- | --- | --- | --- |
| IgG | SERS | 10 fg/mL | 5 |
| Her2 | SERS | 10 fg/mL | 6 |
| PSA | SERS | 12 pg/mL | 7 |
| IgG | Fluorescence | 150 pg/mL | 8 |
| KIM1 | Fluorescence | 500 pg/mL | 9 |
| BNP | Fluorescence | 74.5 pg/mL | 10 |
| TNF-α | SPR | 0.5 ng/mL | 11 |
| CEA | ECL | 530 pg/mL | 12 |
| IgG | DFM | 1 pg/mL | 2 |
| AFP | SERS | 100 fg/mL | This work |
| PSA | SERS | 180 fg/mL |  |

IgG: immunoglobulin G; Her2: human epidermal growth factor receptor 2; PSA: prostate specific antigen; KIM1: kidney injury molecule-1; BNP: B-type natriuretic peptide; TNF-α: tumor necrosis factor alpha; CEA: carcinoembryonic antigen; AFP: alpha fetoprotein; SPR: surface plasmon resonance; ECL: electrochemiluminescent; DFM: dark-field microscopy.

**Section 10 Detection of prostate specific antigen based on SERS**

To verify the universality of this plasmonic sensor, we also detected prostate specific antigen. The SERS spectra and the plot of the SERS intensity as a function of the concentration are shown in Figure S10.


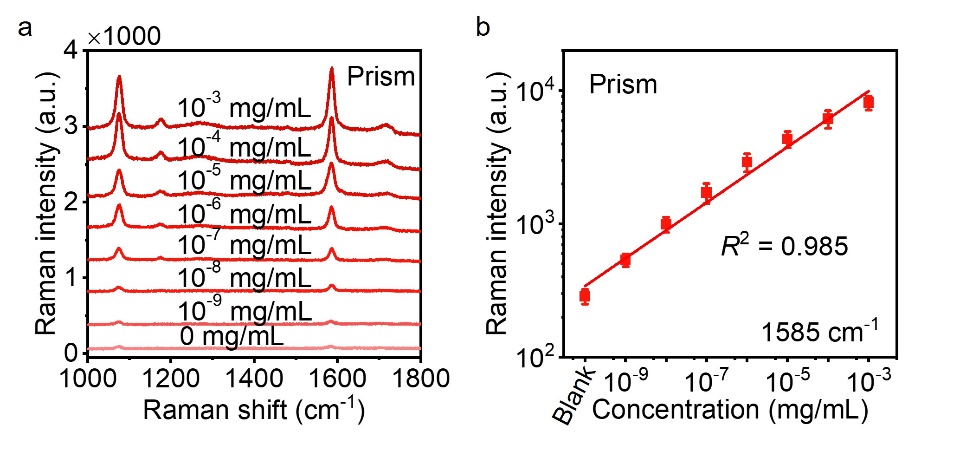


Figure S10 (a) SERS spectra of the immune NPOM substrates with the concentration of prostate specific antigen ranging from 10^-3^ to 10^-9^ mg/mL under prism excitation. (b) Plots of SERS intensities at 1585 cm^-1^ as a function of the concentration of prostate specific antigen.

**Section 11 Quantify AFP by ELISA method**

ELISA is a commonly method in biomarker detection, which is called the “gold standard” in medicine.


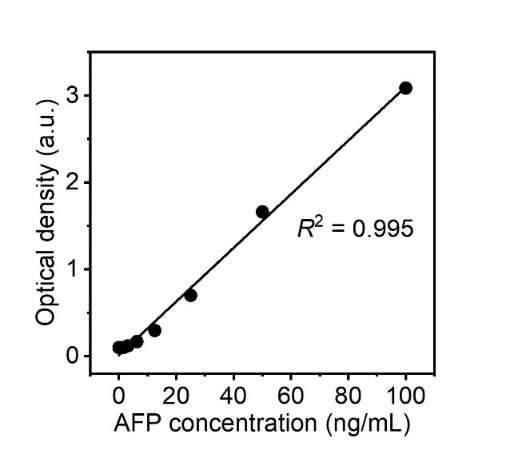


Figure S11 AFP concentration calibration curve obtained by ELISA.

**Section 12 AuNPs distribution density of the NPOM substrate prepared by self-assembly**

The distribution density of NPs on the gold film can be controlled by the adsorption time of NPs on the gold film.


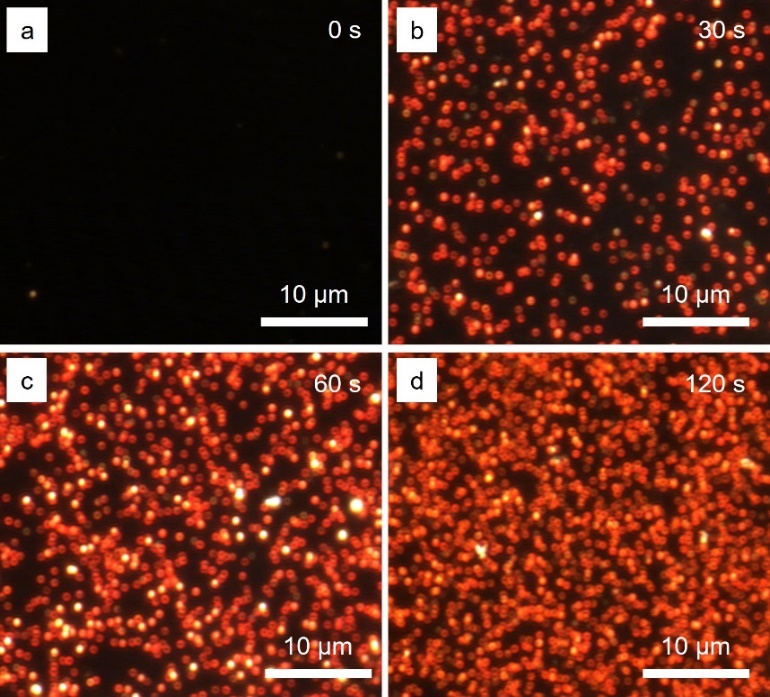


Figure S12 Dark-field images of the substrate under AuNPs solution absorbed on the gold film with different time. (a) A clean gold film without AuNPs absorbed on it. (b-d) NPOM substrate with AuNPs solution absorbed on gold film for 30 s (b), 60 s (c), and 120 s (d).

**References**

(1) Srivastava, S. K.; Li, A.; Li, S.; Abdulhalim, I., Optimal Interparticle Gap for Ultrahigh Field Enhancement by Lsp Excitation Via Esps and Confirmation Using Sers. J. Phys. Chem. C 2016, 120 (50), 28735-28742.

(2) Xiong, Y.; Fu, T.; Zhang, D.; Zhang, S.; Xu, H., Superradiative Plasmonic Nanoantenna Biosensors Enable Sensitive Immunoassay Using the Naked Eye. Nanoscale 2021, 13 (4), 2429-2435.

(3) Qian, X.; Peng, X.-H.; Ansari, D. O.; Yin-Goen, Q.; Chen, G. Z.; Shin, D. M.; Yang, L.; Young, A. N.; Wang, M. D.; Nie, S., In Vivo Tumor Targeting and Spectroscopic Detection with Surface-Enhanced Raman Nanoparticle Tags. Nat. Biotechnol. 2008, 26 (1), 83-90.

(4) Johnson, P. B.; Christy, R. W., Optical Constants of the Noble Metals. Phys. Rev. B 1972, 6 (12), 4370-4379.

(5) Wu, L.; Wang, Z.; Fan, K.; Zong, S.; Cui, Y., A Sers-Assisted 3d Barcode Chip for High-Throughput Biosensing. Small 2015, 11 (23), 2798-806.

(6) Wang, Y.; Vaidyanathan, R.; Shiddiky, M. J. A.; Trau, M., Enabling Rapid and Specific Surface-Enhanced Raman Scattering Immunoassay Using Nanoscaled Surface Shear Forces. ACS Nano 2015, 9 (6), 6354-6362.

(7) Cheng, Z.; Choi, N.; Wang, R.; Lee, S.; Moon, K. C.; Yoon, S.-Y.; Chen, L.; Choo, J., Simultaneous Detection of Dual Prostate Specific Antigens Using Surface-Enhanced Raman Scattering-Based Immunoassay for Accurate Diagnosis of Prostate Cancer. ACS Nano 2017, 11 (5), 4926-4933.

(8) Wang, Z.; Zong, S.; Li, W.; Wang, C.; Xu, S.; Chen, H.; Cui, Y., Sers-Fluorescence Joint Spectral Encoding Using Organic-Metal-Qd Hybrid Nanoparticles with a Huge Encoding Capacity for High-Throughput Biodetection: Putting Theory into Practice. J. Am. Chem. Soc. 2012, 134 (6), 2993-3000.

(9) Luan, J.; Morrissey, J. J.; Wang, Z.; Derami, H. G.; Liu, K. K.; Cao, S.; Jiang, Q.; Wang, C.; Kharasch, E. D.; Naik, R. R.; Singamaneni, S., Add-on Plasmonic Patch as a Universal Fluorescence Enhancer. Light Sci. Appl. 2018, 7, 29.

(10) Cruz, D. F.; Fontes, C. M.; Semeniak, D.; Huang, J.; Hucknall, A.; Chilkoti, A.; Mikkelsen, M. H., Ultrabright Fluorescence Readout of an Inkjet-Printed Immunoassay Using Plasmonic Nanogap Cavities. Nano Lett. 2020, 20 (6), 4330-4336.

(11) Law, W.-C.; Yong, K.-T.; Baev, A.; Prasad, P. N., Sensitivity Improved Surface Plasmon Resonance Biosensor for Cancer Biomarker Detection Based on Plasmonic Enhancement. ACS Nano 2011, 5 (6), 4858-4864.

(12) Khan, M. S.; Ameer, H.; Chi, Y., Label-Free and Ultrasensitive Electrochemiluminescent Immunosensor Based on Novel Luminophores of Ce2sn2o7 Nanocubes. Anal. Chem. 2021, 93 (7), 3618-3625.
